# Up-regulation of hypoxia inducible factor-1α by cobalt chloride correlates with proliferation and apoptosis in PC-2 cells

**DOI:** 10.1186/1756-9966-31-28

**Published:** 2012-03-27

**Authors:** Zhi-Jun Dai, Jie Gao, Xiao-Bin Ma, Kun Yan, Xiao-Xu Liu, Hua-Feng Kang, Zong-Zheng Ji, Hai-Tao Guan, Xi-Jing Wang

**Affiliations:** 1 Department of Oncology, the Second Affiliated Hospital, Medical School of Xi'an Jiaotong University, Xi'an, China; 2 Department of Nephrology, the Second Affiliated Hospital, Medical School of Xi'an Jiaotong University, Xi'an, China; 3 Department of General Surgery, the Second Affiliated Hospital, Medical School of Xi'an Jiaotong University, Xi'an, China

**Keywords:** Pancreatic carcinoma, Hypoxia, Cobalt chloride, HIF-1α, Apoptosis, Proliferation

## Abstract

**Background:**

The exact mechanism of the effects of hypoxia on the proliferation and apoptosis in carcinoma cells is still conflicting. This study investigated the variation of hypoxia-inducible factor-1α(HIF-1α) expression and the apoptosis effect of hypoxia stimulated by cobalt chloride (CoCl_2_) in pancreatic cancer PC-2 cells.

**Methods:**

PC-2 cells were cultured with different concentration (50-200 μmol/L) of CoCl_2 _after 24-120 hours to simulate hypoxia in vitro. The proliferation of PC-2 cells was examined by MTT assay. The cellular morphology of PC-2 cells were observed by light inverted microscope and transmission electron microscope(EM). The expression of HIF-1α on mRNA and protein level was measured by semi-quantitive RT-PCR and Western blot analysis. Apoptosis of PC-2 cells were demonstrated by flow cytometry with Annexin V-FITC/PI double staining.

**Results:**

MTT assay showed that the proliferation of PC-2 cells were stimulated in the first 72 h, while after treated over 72 h, a dose- dependent inhibition of cell growth could be observed. By using transmission electron microscope, swollen chondrosomes, accumulated chromatin under the nuclear membrane and apoptosis bodies were observed. Flow cytometer(FCM) analysis showed the apoptosis rate was correlated with the dosage of CoCl_2_. RT-PCR and Western blot analysis indicated that hypoxia could up-regulate the expression of HIF-1α on both mRNA and protein levels.

**Conclusion:**

Hypoxic microenvironment stimulated by CoCl_2 _could effectively induce apoptosis and influence cell proliferation in PC-2 cells, the mechanism could be related to up-expression of HIF-1α.

## Background

Hypoxia is one of the most important pathological characteristics of solid tumor which is the result of imbalance between tumor cell proliferation and blood supply [1]. As solid tumor growing, its center becomes a hypoxic area because of lacking blood and oxygen. The hypoxic status of various solid tumor has been recognized as an important determinant for the outcome of anti-cancer therapies in a number of tumors [2].

Hypoxia-inducible factor-1 (HIF-1) was found in the 1992 when Semenza [3] researched the expression of erythropoietin gene induced by hypoxia. Human HIF-1 has been depurated and isolated, it is a heterodimeric transcription factor composed of oxygen-dependent HIF-1α and constitutively expressed HIF-1β subunits, HIF-1 transcriptional activity is largely determined by regulated expression of the HIF-1α subunit [4]. HIF-1α over-expression has been detected in various tumors including breast, oropharyngeal, nasopharyngeal, prostate, brain, lung, stomach cancer and so on, and has been associated with tumor aggressiveness, vascularity, treatment failure and mortality [5-7]. Interestingly, HIF-1α can also over-expressed under normoxic conditions in some human tumors [8].

In this research, we treated a human pancreatic cancer cell line (PC-2) with cobalt chloride (CoCl_2_) to stimulate hypoxia *in vitro*. Under the hypoxic condition, we observed the proliferation of PC-2 cells by MTT assay. Meanwhile, RT-PCR and Western blot analysis were conducted to measure the expression of HIF-1α on mRNA and protein level. Furthermore, we discussed the effect of hypoxic microenvironment on apoptosis and its mechanism.

## Materials and methods

### Reagents

Fetal bovine serum (Gibco, USA); RPMI1640 medium (Gibco, USA); 3-(4,5) -dimethylthiahiazo(-z-y1)-3,5- diphenyte- trazoliumromide (MTT) (Gibco, USA); annexin V-FITC/PI apoptosis detection kit (Becon Dickinson Facsalibur, USA); RT-PCR kit (ampliqon, Denmark); Trizol (Invitrogen, USA); HIF-1α monoclonal antibody (Santa Cruz Biotechnology, USA); 3-(5'-hydroxymethyl-2'-furyl)-1 -benzylindazole (YC-1) (Shanghai Shenggong Biological Engineering Technology&Service, China); CoCl_2 _(Shanghai Shenggong Biological Engineering Technology&Service, China).

### Cell line and cell culture

Human pancreatic cancer cell line, PC-2, was purchased from the medical experimental animal center of the fourth military medical university. Cells were cultured in RPMI 1640 maximal medium containing 10% inactived fetal bovine serum (56°C, 30 min), 1 × 10^5 ^U/L penicillin and 100 mg/L streptomycin in a humidified atmosphere with 5% CO_2 _incubator at 37°C.

### MTT assay for the proliferation of PC-2 cells

The proliferation of PC-2 cells was assessed using MTT dye reduction assay (Sigma, USA), which was conducted as described previously [9]. PC-2 cells were seeded in a 96-well plate at a density of 1 × 10^4 ^cells/well, cultured for 12 h under 37°C in 5% CO_2_, then treated with different concentration (50, 100, 150, 200 μmol/L) CoCl_2 _for 24-120 h. At the end of the treatment, MTT, 50 μg/10 μL, was added and the cells were incubated for another 4 hours. Dimethylsufloxide (DMSO; 200 μl) was added to each well after removal of the supernatant. After shaking the plate for 10 min, cell viability was assessed by measuring the absorbance at 490 nm using an Enzyme-labeling instrument (EX-800 type); all measurements were performed three times. Cell growth curve was completed using time as the abscissa and *A *value (mean ± SD) as the ordinate.

### Detection of morphological change by transmission electron microscope

Uranyl acetate and lead citrate staining of cells were performed to detect morphological changes. Briefly, adherent PC-2 cells were treated with 200 μmol/L CoCl_2 _for 48 hours. After treatment, the treated cells were digested with pancreatin and fixed with 3% glutaraldehyde precooled in 4°C for 2 hours. To make ultra-thin sections of copper, cells were washed with phoisphate-buffered salein (PBS) once, fixed with 1% osmic acid for 1 hour, dehydrated by acetone and embedded in epoxide resin. After staining with uranyl acetate and lead citrate, the sections were examined by a Hitachi-800 transmission electron microscope [10].

### Semi-quantitative reverse transcription polymerase chain reaction (RT-PCR) assay

PC-2 cells were seeded in 6 cm culture capsules and treated with concentration gradient CoCl_2 _(0, 50, 100, 150, 200 μmol/L) separately for 8 h. In the group of 200 μmol/L, we selected cells at 0 h, 4 h, 8 h and 12 h point for further experiment. And then treated with 2.0 μmol/L YC-1 (0, 50, 100, 150,) for 2 h. As previously described [11], cells collected at specified time were used to extract total RNA using the Trizol reagent following the manufacturer's instructions. 1 μgRNA synthetized cDNA through reverse transcriptase undergo listed below condition: 70°C 5 min, 42°C extended for 60 min, 95°C enzyme inactivated for 3 min and 4°C terminated reaction. Synthetical cDNA as template to carry out polymerase chain reaction. HIF-1α primer sequence (Invitrogen CO): 5'-ACTTCTGGATGCTGGTGATT-3' (sense) and 5'-TCCTCGGCTAGTTAG GGTAC -3' (anti-sense), amplification fragment was 325 bp, renaturation temperature was 55°C (cycling 35 times). β-actin, its primer sequence was 5'-GTTGCGTTACACCCTTTCTTG-3' (sense), 5'-TGCTGTCACCTTCACCGT TC-3' (anti-sense), amplification fragment was 133 bp, and renaturation temperature was 55°C (cycling 40 times). Amplification condition was below: pre-denaturized for 3 min at 95°C, denaturized for 30s at 95°C, renaturated for 30s at 55°C and extended for 30s at 72°C. PCR product was detected on agarose gel electrophoresis and ethidium bromide imaging system was used to make density index analysis. The expression intensity of HIF-1α mRNA was denoted with the ratio of the photodensity of the RT-PCR products of HIF-1α and β-actin.

### Western blot analysis

As previously described [12], cells were washed with ice-cold PBS twice and lysed with lysis buffer containing 1% NP40, 137 mM NaCL, 20 mM Tris base(pH7.4), 1 mM DTT, 10% glycerol, 10 mg/mL Aprotinin, 2 mM sodium vanadate and 100 μM PMSF. Protein concentrations were determined using the PIERCE BCA protein assay kit. Protein was separated by 10% SDS-PAGE under denaturing conditions and transferred to nitrocellulose membranes. Membranes were incubated with an mouse HIF-1α monoclonal antibody (1:1000; Santa Cruz Biotechnology), followed by incubation in goat antimouse secondary antibody conjugated with horseradish peroxidase (1:1000; Santa Cruz Biotechnology). Immunoreactive proteins were visualized using enhanced chemiluminescence detection system (Amersham Biosciences)

### Apoptosis detection by FCM

Apoptotic cells were differentiated from viable or necrotic ones by combined application of annexin V-FITC and propidium iodide (PI) (BD Biosciences Clontech, USA) [13]. The samples were washed twice and adjusted to a concentration of 1 × 10^6 ^cells/mL with 4°C PBS. The Falcon tubes (12 mm × 75 mm, polystyrene round-bottom) were used in this experiment, 100 μL of suspensions was added to each labeled tube, 10 μL of annexin V-FITC and 10 μL PI(20 μg/mL) were added into the labeled tube, incubated for at least 20 min at room temperature in the dark, then 400 μL of PBS binding buffer was added to each tube without washing and analyzed using FCM analysis (BD Biosciences Clontech, USA) as soon as possible (within 30 min). This assay was done quintuplicate.

### Statistical analysis

All data were expressed by mean ± S.E.M. Statistical analyses were performed using SPSS 11.0 for Windows software. ANOVA (one-way analysis of variance) and Student's *t*-test were used to analyze statistical differences between groups under different conditions. *P*-value < 0.05 was considered statistically significant.

## Results

### The influence of hypoxia on PC-2 cells proliferation

**Figure 1:**
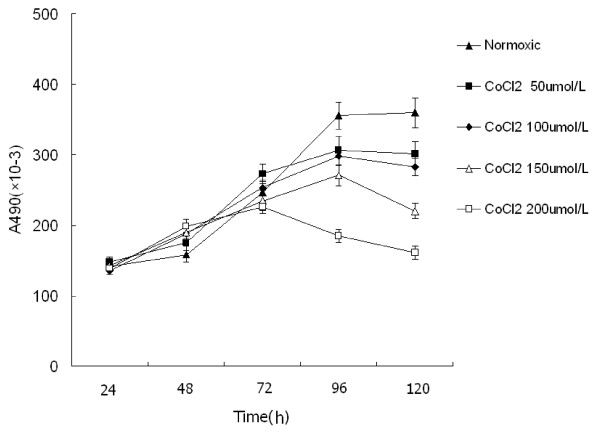
**The growth curve of PC-2 cells treated with different dose of CoCl_2_. Cell viability was determined by MTT method**. This assay was performed in triplicate. Dose- dependent inhibition of cell growth could be observed after 72 h (*P *< 0.05, ANOVA analysis).

We studied the proliferation of PC-2 cells under hypoxia simulated by CoCl2 using MTT assay. As shown in Figure 1, the growth curve of cells under normoxia showed "S" shape: 24-48 h was detention period (slowly grow), 72-96 h were exponential phase of growth (rapidly proliferate), the following 24 h was platform period. Compared with the normoxic group, the cells of hypoxic group didn't show "S" shape. Following a 72 h hypoxic exposure, the proliferation speed of cells under hypoxia was faster, 72 h later, the speed was slower, achieved saturation density in advanced, went into platform period but gradually degraded at 96-120 h. Meanwhile, as the hypoxia became serious, this phenomenon was more conspicuous. After treated over 72 h, a dose- dependent inhibition of cell growth could be observed.

### Morphological changes of PC-2 cells induced by hypoxia

**Figure 2:**
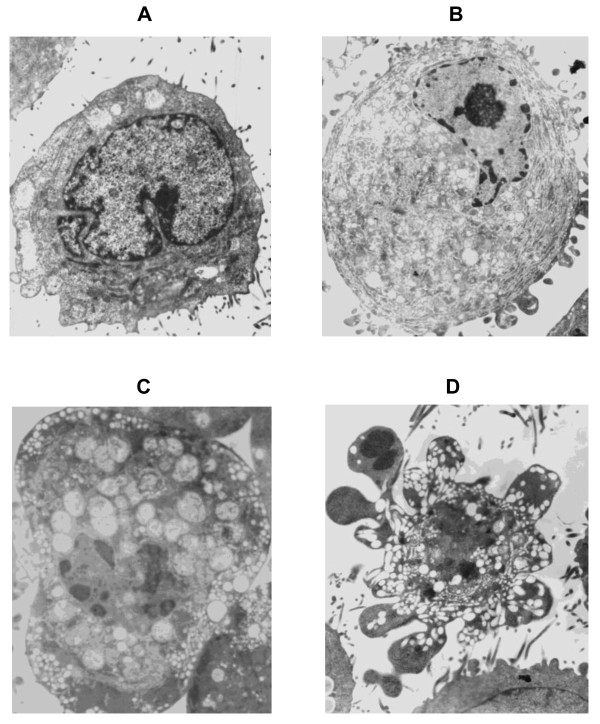
**Morphological changes of PC-2 cells induced by hypoxia by transmission electron microscope**. **A: **Normal pancreatic cancer PC-2 cells(×6000); **B: **PC-2 cells in early stage of apoptosis (×6000); **C: **PC-2 cells in middle stage of apoptosis cell(×6000); **D: **Apoptotic body(×6000).

By using transmission electron microscope, normal PC-2 cells were round and regular, with abundant organelles, the chromatin margination showed in few cells (Figure 2A). After treated with CoCl_2 _for 48 hours, part of nuclear membrane domed outward with a sharp angle. The following different apoptotic periods could be observed. (1) Early stage of apoptosis: the nuclei showed chromatin pyknosis, and were clustered on the inner border of karyotheca; cytoplasm condensation and swelling of mitochondria were observed in the inner segment; the nucleus was at one end of the cell with complete karyotheca and many mitochondria in the cytoplasm showed the early ultrastructure changes of apoptosis (Figure 2B). (2) Middle stage of apoptosis: in addition to the swelling of mitochondria and many vacuoles, the surface of cellular membrane process to crassitude, and the endoplasmic reticulum was abundant; the typical changes were karyopyknosis or karyorrhexis (Figure 2C). (3) Late stage of apoptosis: characterized by changes such as shrinkage, condensation of nuclear chromatin, fragmentation of nuclei and formation of apoptotic bodies (showed in Figure 2D)

### Expression of HIF-1α mRNA detected by semi-quantitive RT-PCR

**Figure 3:**
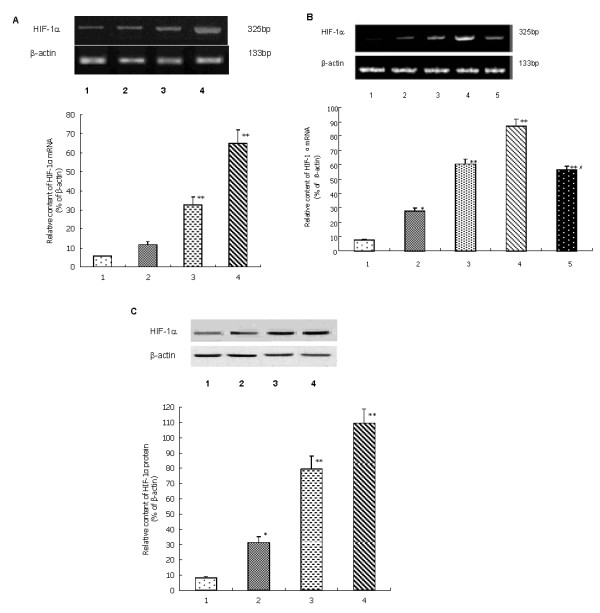
**A: The expression of HIF-1α mRNA in PC-2 cells treated with different concentration of CoCl_2_**. **1**. Normoxia group; **2**. CoCl_2 _100 μmol/L group; **3**. CoCl_2 _150 μmol/L group; **4**. CoCl_2 _200 μmol/L group. This assay was done quintuplicate. Values represent means ± standard deviations (*n *= 5) and were determined using the Student's *t*-test. *P < 0.05 and **P < 0.01 versus Normoxia group. **B: **The expression of HIF-1α mRNA in PC-2 cells treated with 200 μmol/L CoCl_2 _for different time. **1**. 0 h; **2**. 4 h; **3**. 8 h; **4**. 12 h; **5**. YC-1 2 h. This assay was done quintuplicate. Values represent means ± standard deviations (*n *= 5) and were determined using the Student's *t*-test. *P < 0.05, **P < 0.01 versus 0h, ^#^*P *< 0.05 versus 12h. **C: **The expression of HIF-1α protein in PC-2 cells treated with different concentration of CoCl_2_. **1**. Normoxia group; **2**. CoCl_2 _100 μmol/L group; **3**. CoCl_2 _150 μmol/L group; **4**. CoCl_2 _200 μmol/L group. This assay was done quintuplicate. Values represent means ± standard deviations (*n *= 5) and were determined using the Student's *t*-test. *P < 0.05 and **P < 0.01 versus Normoxia group.

RT-PCR revealed HIF-1α mRNA expressed rarely in normoxic PC-2 cells, as CoCl_2 _density increased its expression gradually increased (Figure 3A). When cells treated with 200 μmol/L CoCl_2_, accompanied with the action time extended the expression of HIF-1αmRNA increased (Figure 3B). The correlation of CoCl_2 _and HIF-1α mRNA was a dose- and time-dependent manner. After treated with YC-1 for 2 h, overexpression of HIF-1αmRNA induced by CoCl_2 _was significantly down-regulated (Figure 3B).

### Expression of HIF-1α protein detected by western blot analysis

The protein level of HIF-1α was measured in PC-2 cells treated with different doses of CoCl_2 _by Western blot analysis employing mouse monoclonal HIF-1α antibodies. As shown in Figure 3C, the amount of HIF-1α protein after CoCl_2 _treatment was significantly increased in a dose-dependent manner (*P *< 0.05). These data demonstrated that hypoxic microenvironment simulanted by CoCl_2 _could up-regulate HIF-1α expression.

### FCM analysis of cell apoptosis induced by hypoxia

**Figure 4:**
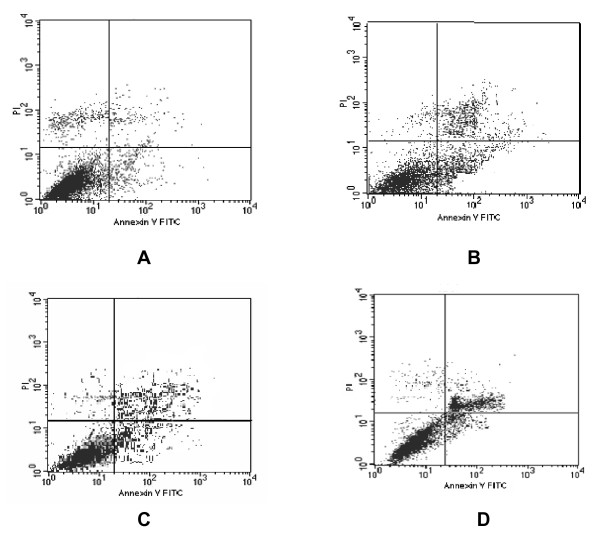
**Flow cytometry was used to observe the apoptosis of PC-2 cells by staining with annexinV-FITC/PI**. **A**. Normoxia group; **B**. CoCl_2 _100 μmol/L group; **C**. CoCl_2 _150 μmol/L group; **D**. CoCl_2 _200 μmol/L group.

After treatment with different doses of CoCl_2 _for 72 h, apoptosis induction was demonstrated using FCM analysis. Apoptotic cells were differentiated from viable or necrotic ones by combined application of annexin V-FITC and PI. Apoptotic and necrotic cells were distinguished according to annexin V-FITC reactivity and PI exclusion. As shown in Figure 4, in normoxic group, there were almost normal cells, rarely viable apoptotic cells; while in hypoxic group, the rate of apoptotic cells was gradually increased along with increasing concentrations of CoCl_2_. The rate of apoptosis in normoxic, 100-200 μmol/L CoCl_2 _group were 10.77%, 34.32%, 40.17%, 52.30%, respectively. Furthermore, apoptotic cells gradually increased in a dose-dependent manner.

## Discussion

More recently, experimental and clinical studies demonstrated that intra-tumor hypoxia might be a key factor in tumor microenvironment promoting invasive growth and metastasis [14]. The increased malignancy of hypoxic tumors has been attributed to the ability of hypoxia to select for cells with diminished apoptotic potential and to induce their clonally expansion [15]. Since the hypoxic phenomenon in tumors was revealed, more and more evidence indicated hypoxia existed in solid tumor generally [16].

Pancreatic cancer is common malignant tumor of digestive system which has high malignancy, difficulty in treatment and poor prognosis. And less than 10% of pancreatic cancer is resectable when being diagnosised and 5-year overall survival rate is less than 5% [17]. During the development of pancreatic cancer, the blood can't supply the tumor nourishment, thus the tumor are hypoxic partly, while hypoxia makes the tumor cell more malignant. In this way, the rapid growth and the hypoxia are unity of opposites in tumors [18].

CoCl_2 _is a chelator which instead of Fe^2+ ^in hemoglobin, and then damage cell's reception of oxygen [19]. The mechanism of CoCl_2 _simulating hypoxia is similar with hypoxic microenvironment *in vivo*, because they have identical signal transduction and transcription regulation. Moreover previous research demonstrated CoCl_2 _correlated with proliferation and apoptosis in human carcinoma cells [20,21]. In our study, we treated PC-2 cells with CoCl_2 _to simulate hypoxic microenvironment, MTT assay revealed along with the increased CoCl_2 _concentration, the exponential phase of PC-2 cells was earlier in advanced and persisted shorter, cells grew slower and went into platform period early(Figure 1). It is reasonable to assume that the step down in PC-2 cell proliferation correlated with the increased hypoxia, hypoxic microenvironment could slow down the speed of tumor growth.

HIF-1α, a transcription factor regulating genes' expression induced by hypoxia, is a key molecular player in the hypoxic response [22]. HIF-1α is generally resided in mammal and human tissue in hypoxic condition, it has been found over-expressed in about 70% tumor [5-7]. Experiment showed that under hypoxic the transcriptive activity of HIF-1α was increasing, which indicated that hypoxic microenvironment might increase the genetic transcriptional level of HIF-1α to regulate the expression of downstream gene [22,23]. However, some scholars presumed hypoxic microenvironment could enhance the stability of HIF-1α [24]. Our present research indicated HIF-1α obviously increased at both protein level and mRNA level in PC-2 cells under hypoxic microenvironment, and it was positive correlated with the hypoxic time and the density of CoCl_2_. This suggested the level of hypoxia was coinciding with the expression of HIF-1α.

Whether HIF-1α can promote tumor cell apoptosis or anti- apoptosis, the opinion didn't reach unify, different research suggest converse results. Some date indicated overexpressed HIF-1α could promote apoptosis by activating Bcl-2 and Bcl-Xl or enhancing the stability of p53 [25]. On the other hand, experiment displayed HIF-1α could up-regulate the VEGF and GLUT1 to make tumor cell resist to apoptosis, inhibition of HIF-1α could promote apoptosis [26]. In our research, under electron microscope, PC-2 cells in hypoxic microenvironment were found in different apoptotic stage (Figure 2A-D), most were in early stage. The FCM analysis showed that the apoptotic rate of normal control group, 100 μmol/L group, 150 μmol/L group and 200 μmol/L group, was 10.77%, 34.32%, 40.17%, 52.30%, respectively. These results were consistent with Luo's research [27].

In conclusion, our study suggested that hypoxic microenvironment can effectively induce apoptosis and influence cell proliferation in PC-2 cells, and the mechanism may be concerned with the up-regulation of HIF-1α.

## Conflicts of interest

The authors declare that they have no competing interests.

## Authors' contributions

DZJ and WXJ designed the research. DZJ, GJ, MXB, YK and KHF performed the experiments throughout this research. LXX, JZZ and GHT contributed to the reagents, and participated in its design and coordination. DZJ and GJ analyzed the data; DZJ and MXB wrote the paper. Co-first authors: DZJ and GJ. All authors have read and approved the final manuscript.
